# Supplementary figures and images for: Identification of key yeast species and microbe–microbe interactions impacting larval growth of Drosophila in the wild
Source: eLife. 2023 Dec 27;12:RP90148. doi: 10.7554/eLife.90148 (PMC10752588; doi:10.7554/eLife.90148)

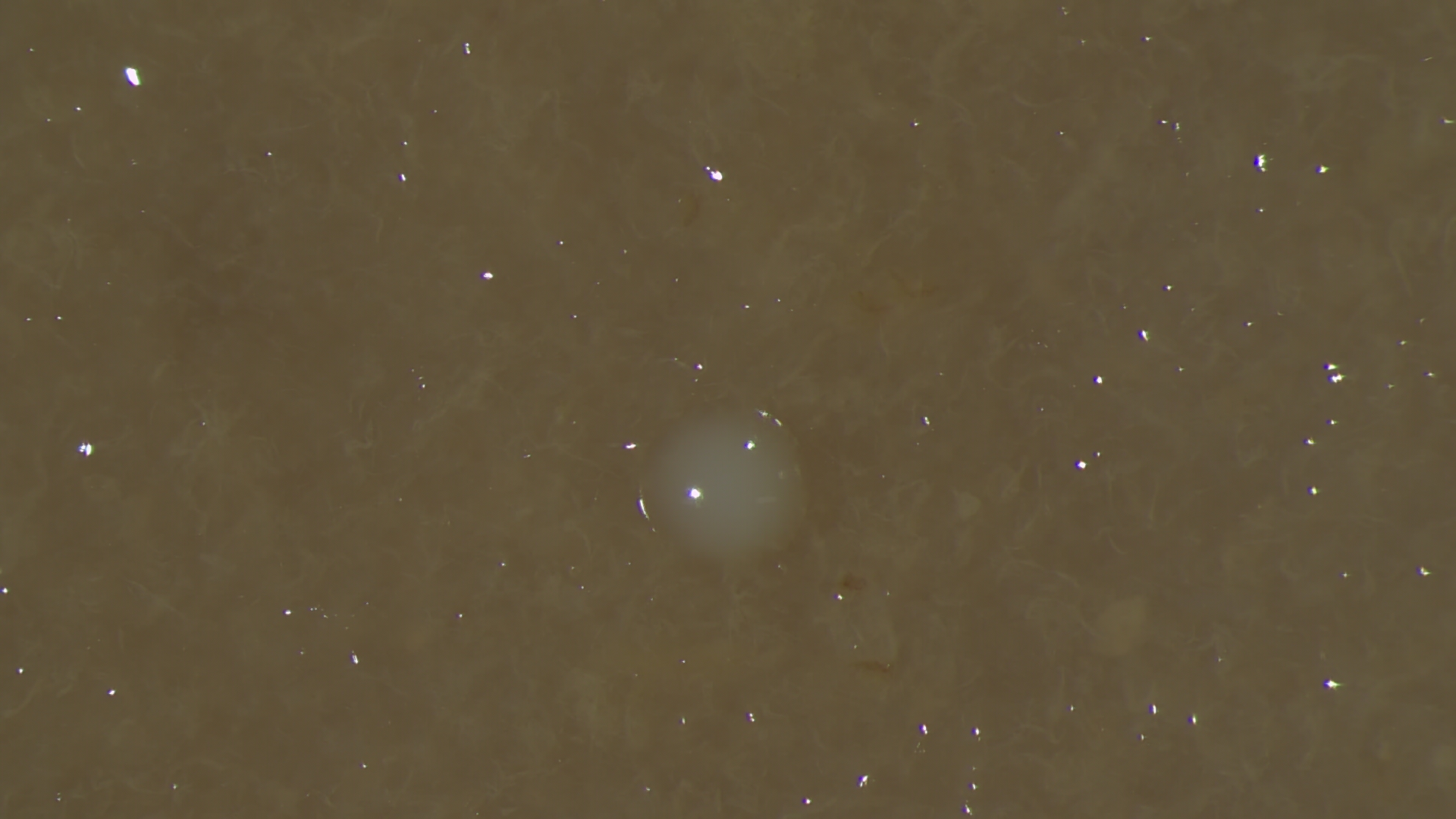

Supplement: Figure 3—figure supplement 1—source data 2. [file elife-90148-fig3-figsupp1-data2.zip › FigureS5_single_colonies_SN/St_bacillaris_banana_agar.jpg]

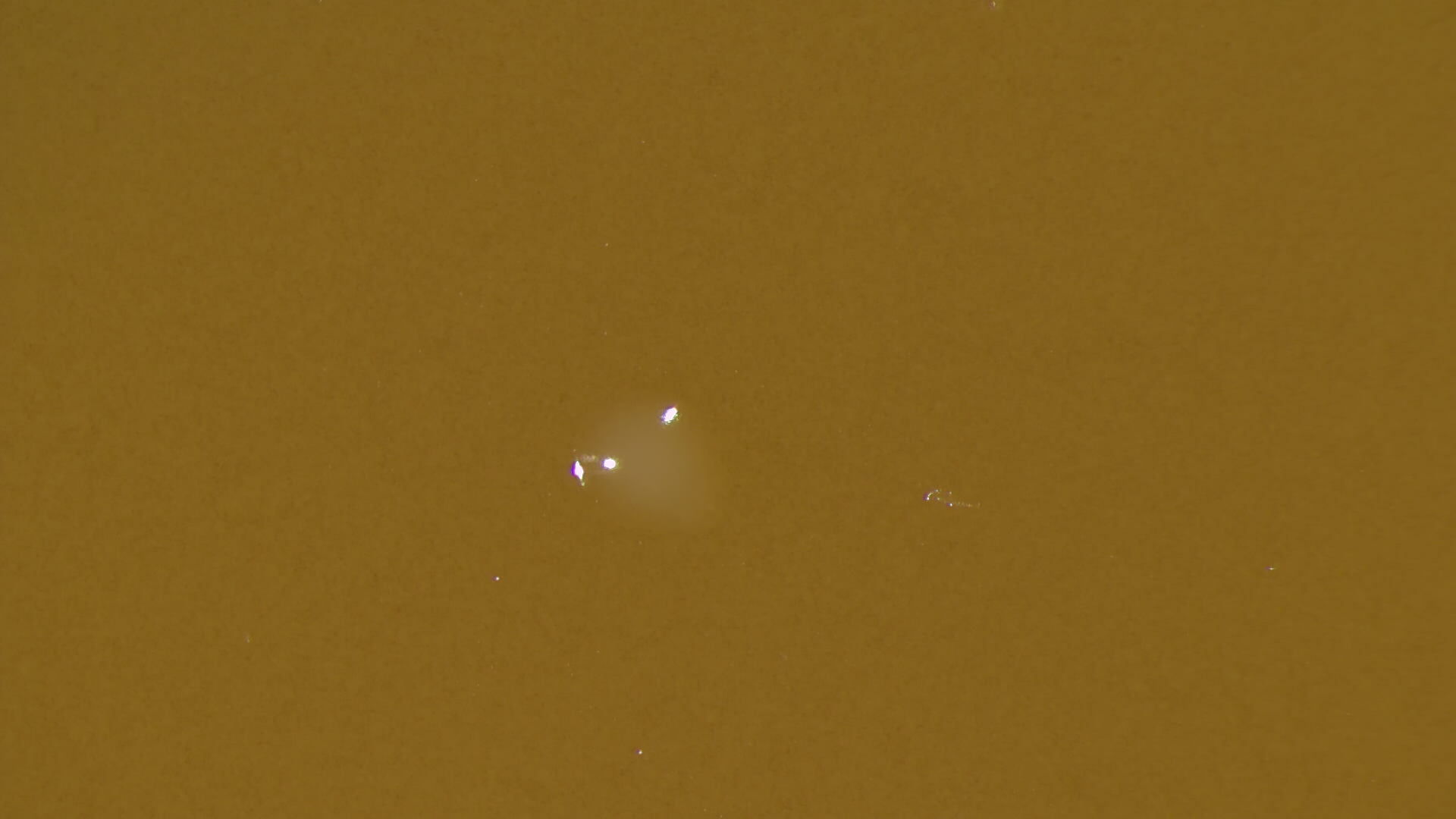

Supplement: Figure 3—figure supplement 1—source data 2. [file elife-90148-fig3-figsupp1-data2.zip › FigureS5_single_colonies_SN/St_bacillaris_nutrient_rich_medium.jpg]

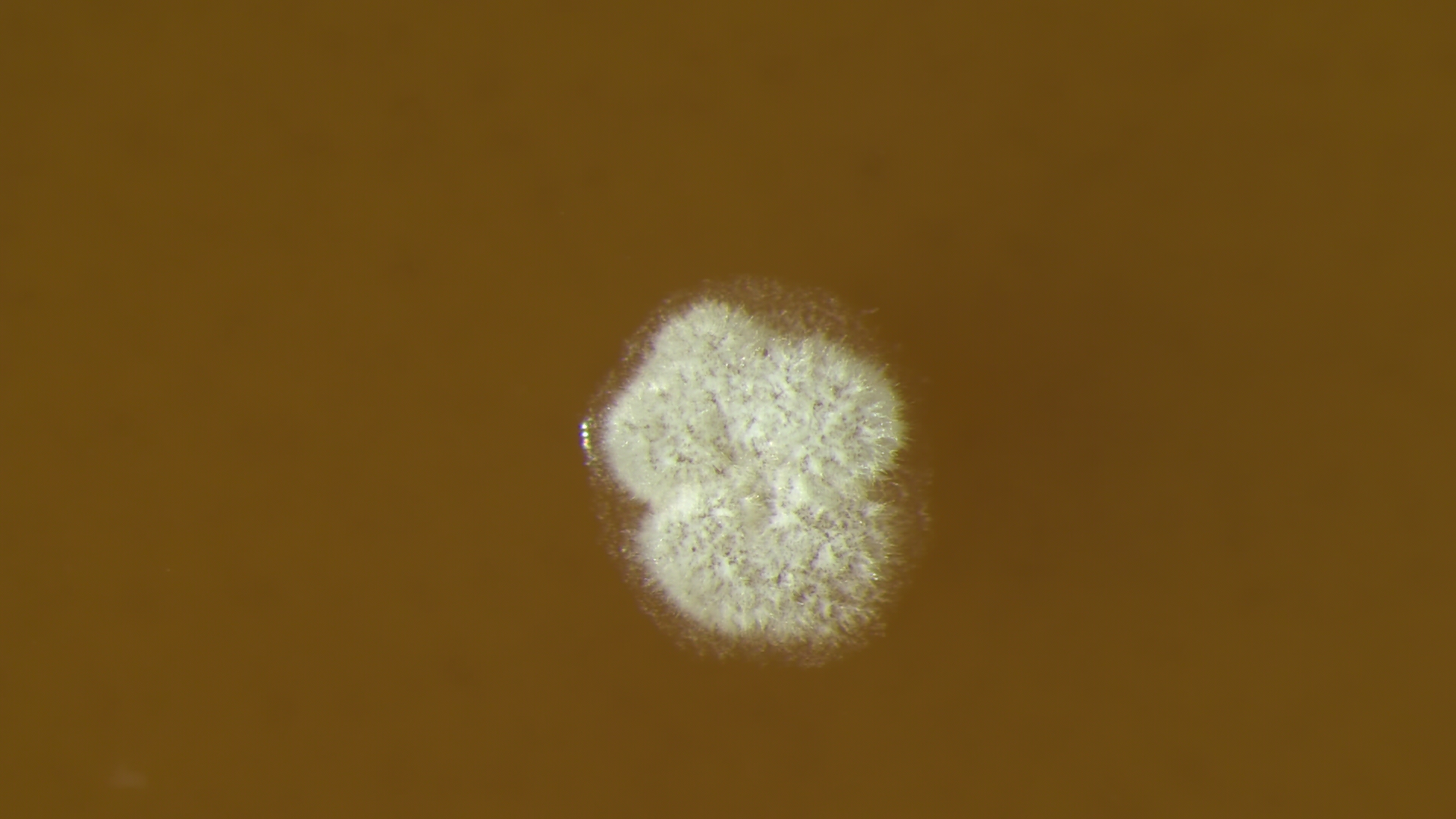

Supplement: Figure 3—figure supplement 1—source data 2. [file elife-90148-fig3-figsupp1-data2.zip › FigureS5_single_colonies_SN/Sa_crataegensis_nutrient_rich_medium.jpg]

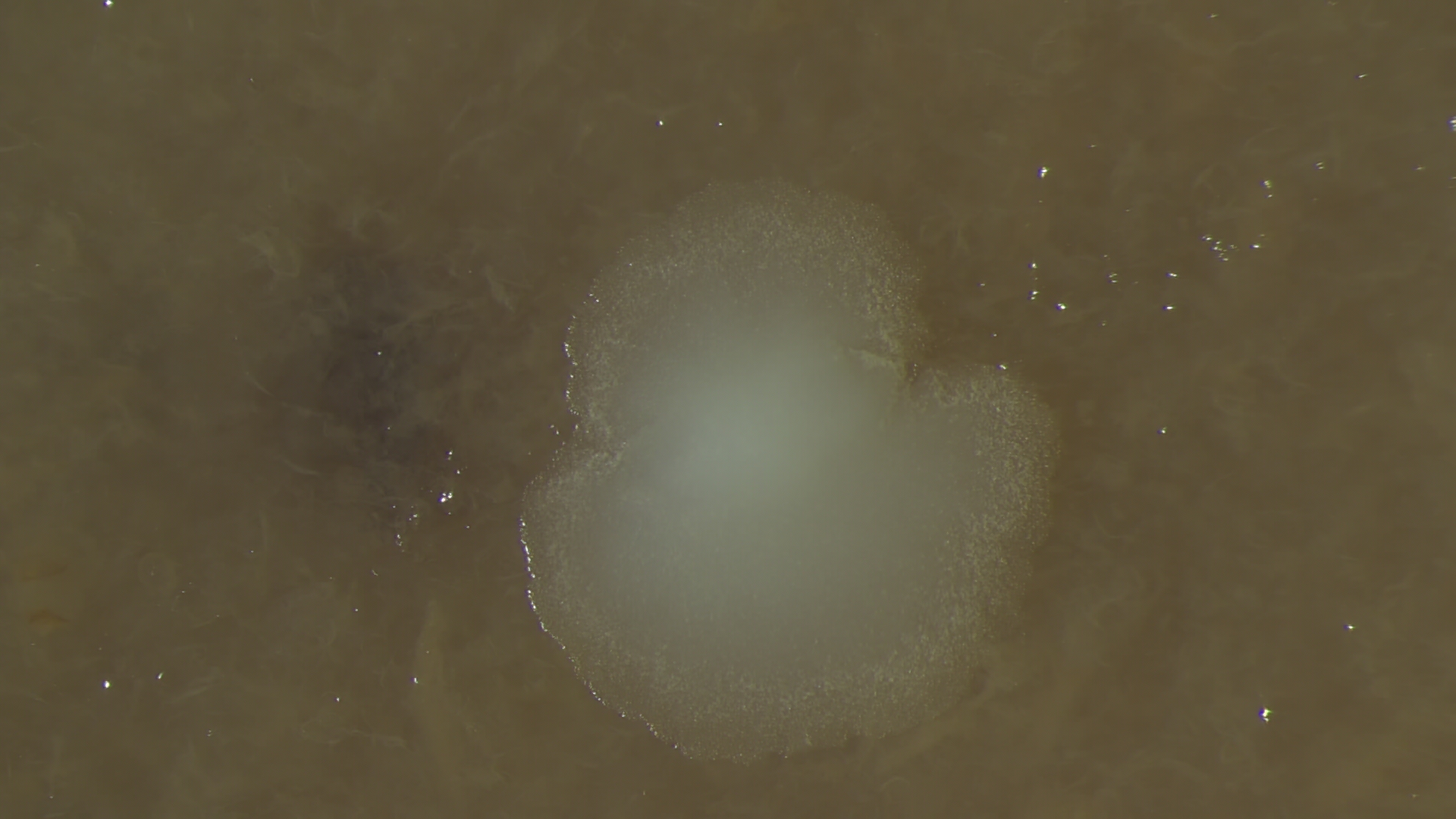

Supplement: Figure 3—figure supplement 1—source data 2. [file elife-90148-fig3-figsupp1-data2.zip › FigureS5_single_colonies_SN/Pi_kluyveri_banana_agar.jpg]

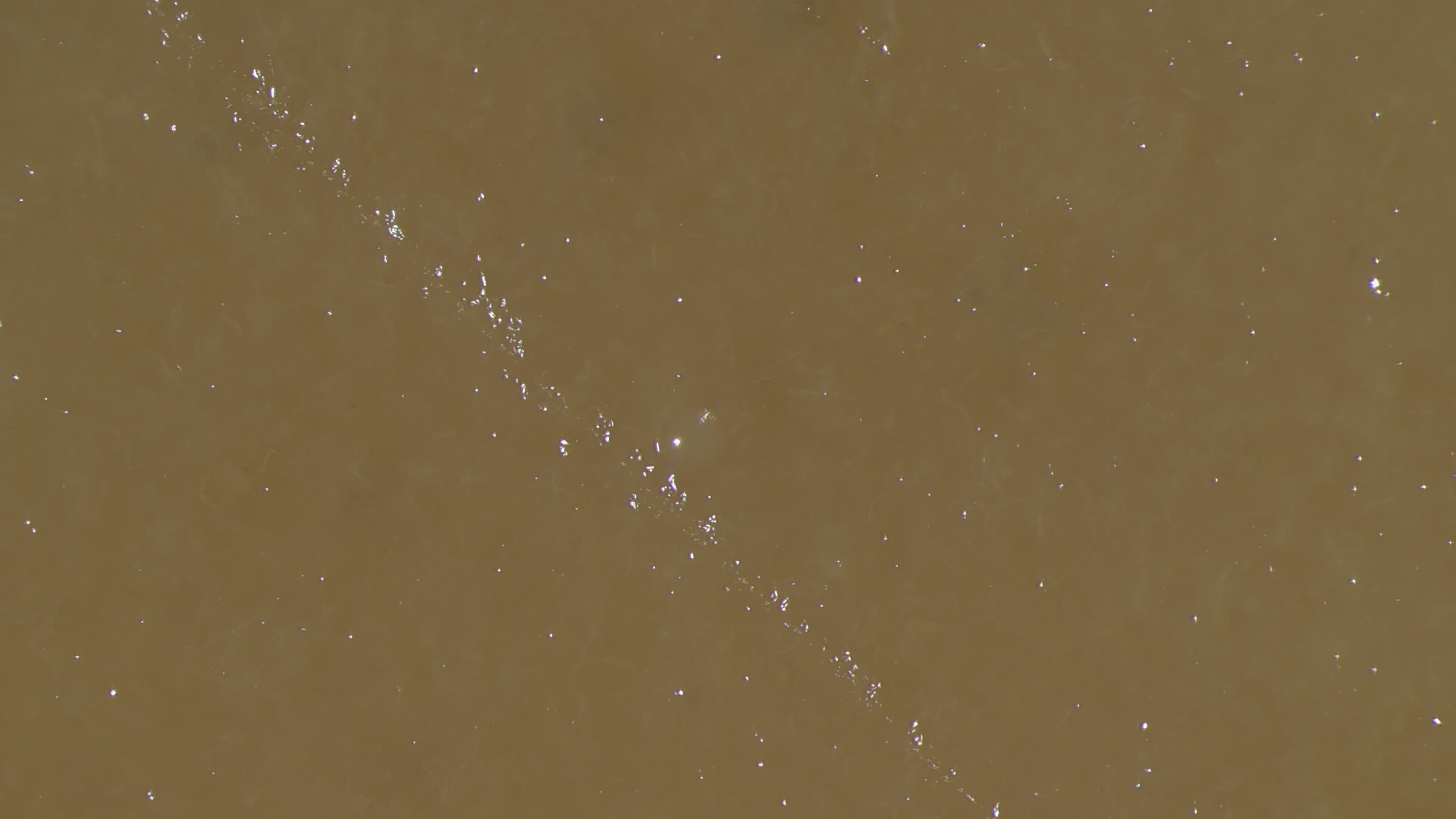

Supplement: Figure 3—figure supplement 1—source data 2. [file elife-90148-fig3-figsupp1-data2.zip › FigureS5_single_colonies_SN/BY4741_banana_agar.jpg]

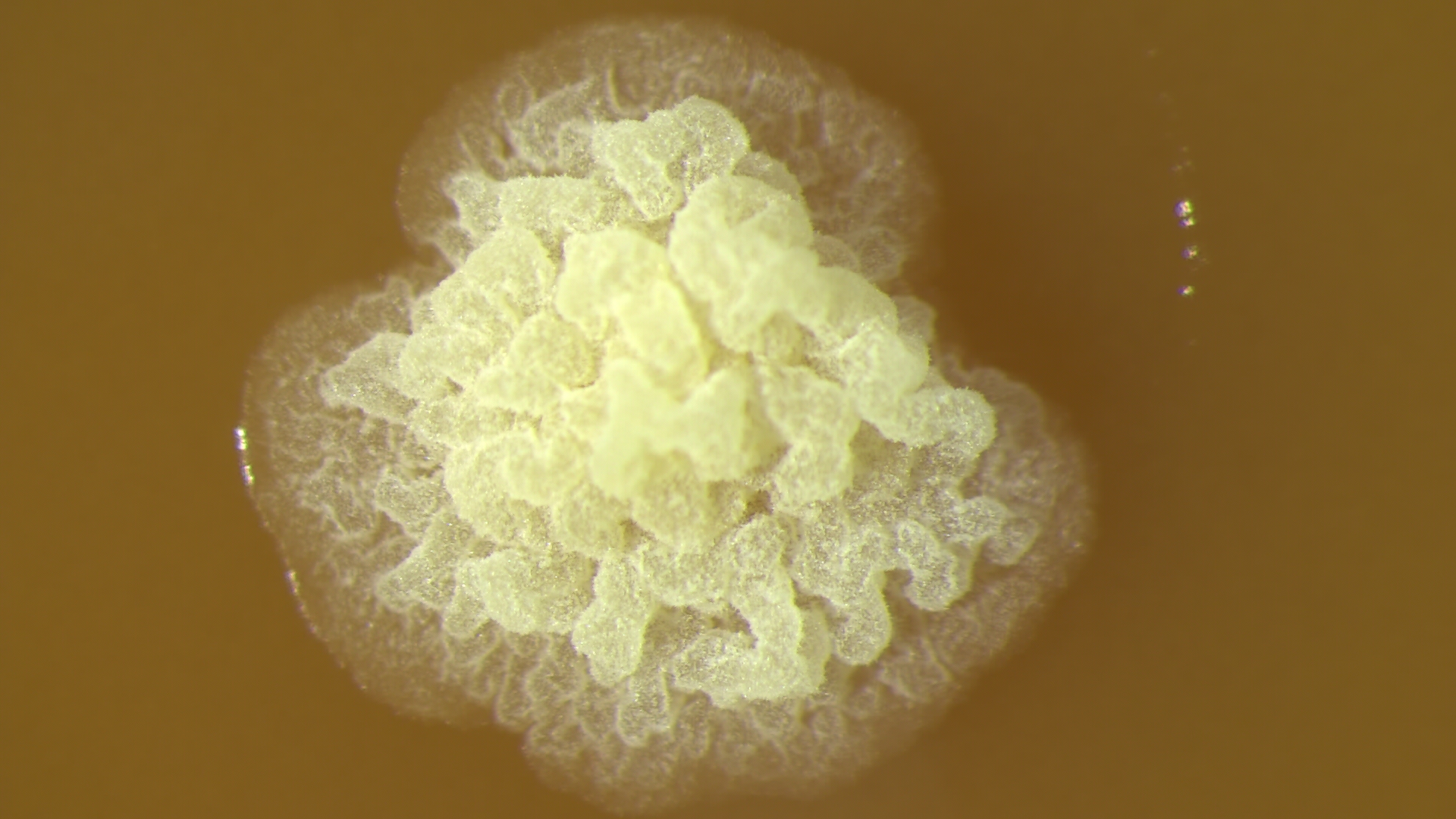

Supplement: Figure 3—figure supplement 1—source data 2. [file elife-90148-fig3-figsupp1-data2.zip › FigureS5_single_colonies_SN/Pi_kluyveri_nutrient_rich_medium.jpg]

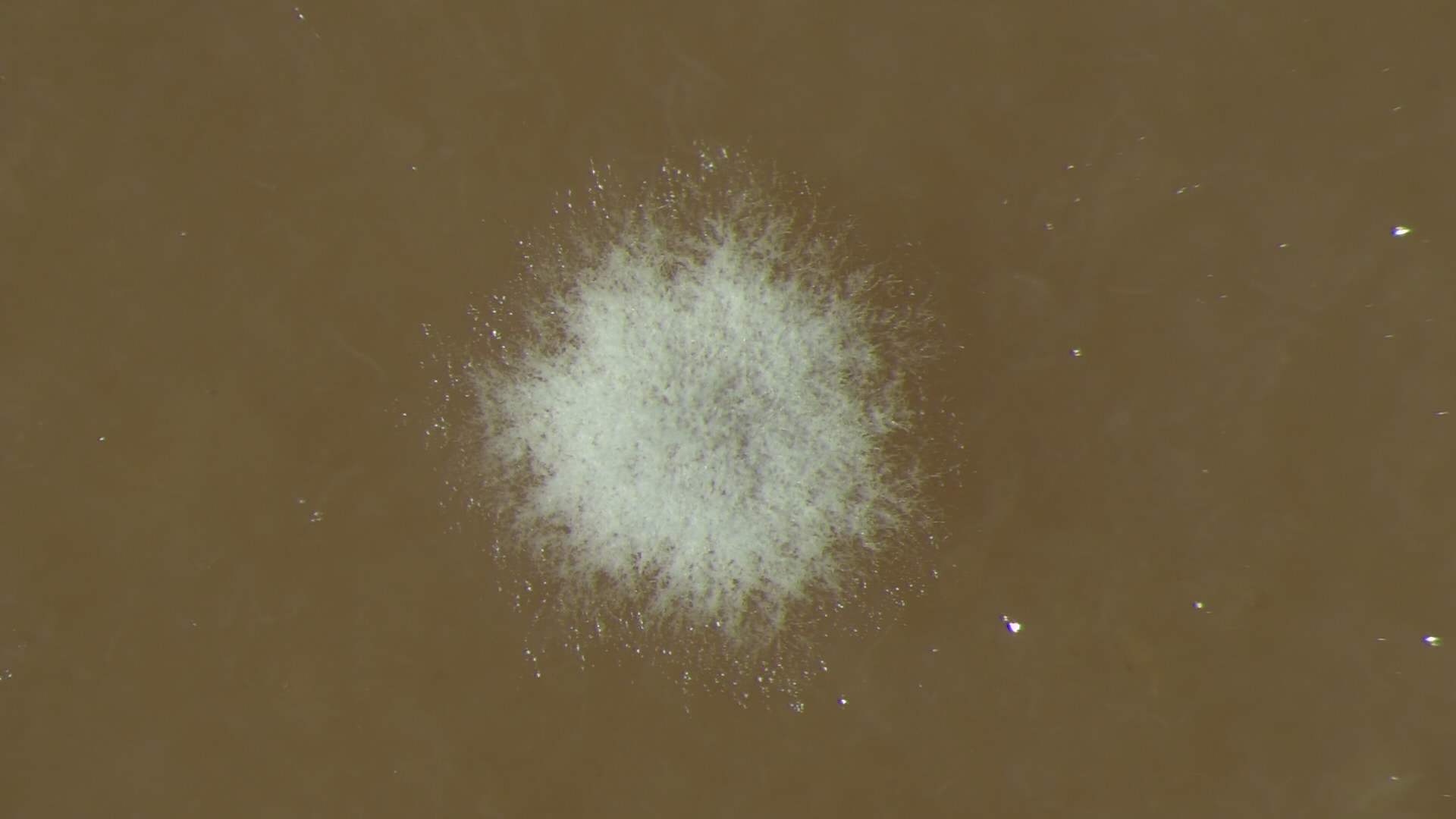

Supplement: Figure 3—figure supplement 1—source data 2. [file elife-90148-fig3-figsupp1-data2.zip › FigureS5_single_colonies_SN/Sa_crataegensis_banana_agar.jpg]

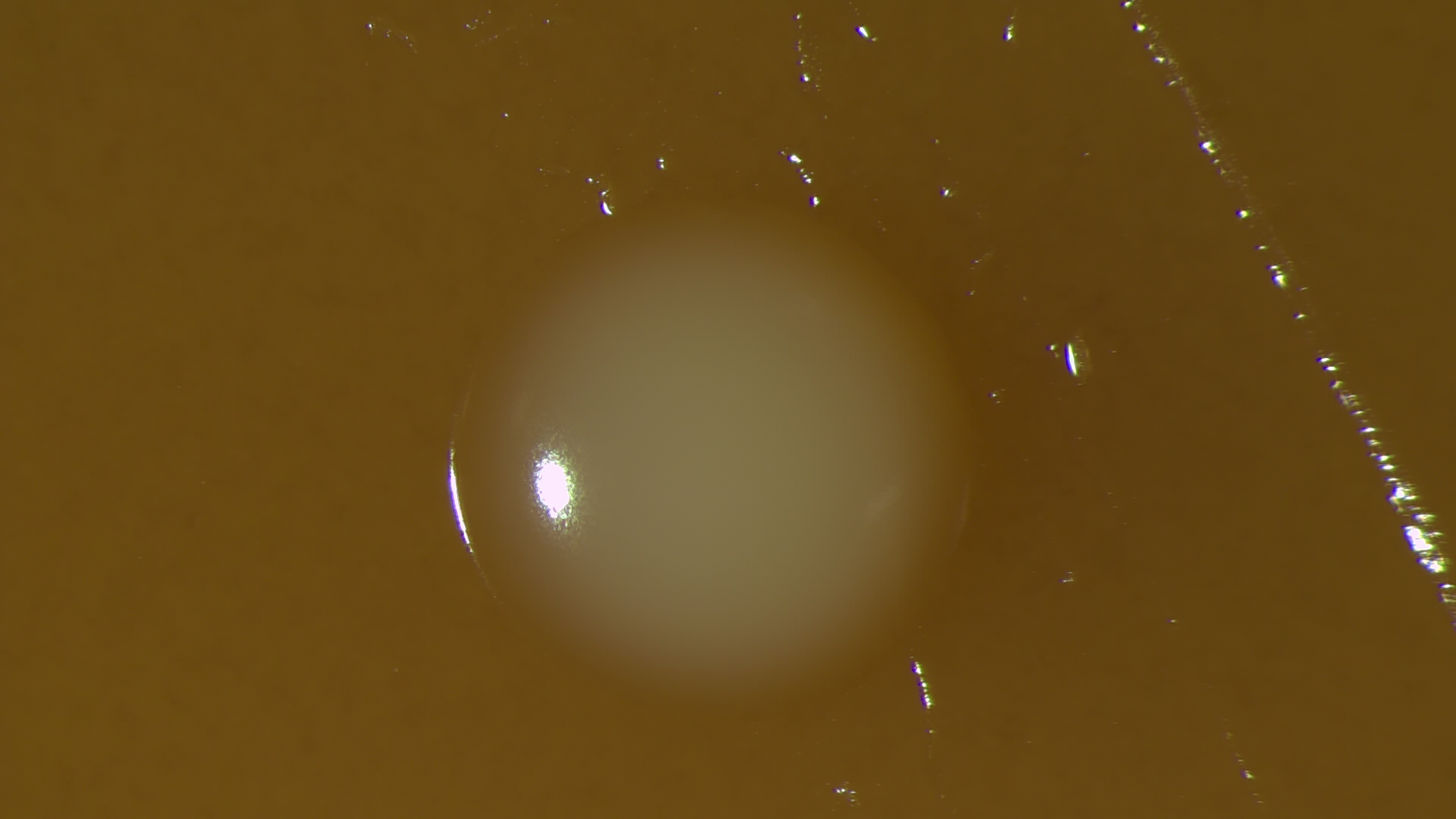

Supplement: Figure 3—figure supplement 1—source data 2. [file elife-90148-fig3-figsupp1-data2.zip › FigureS5_single_colonies_SN/H_uvarum_nutrient_rich_medium.jpg]

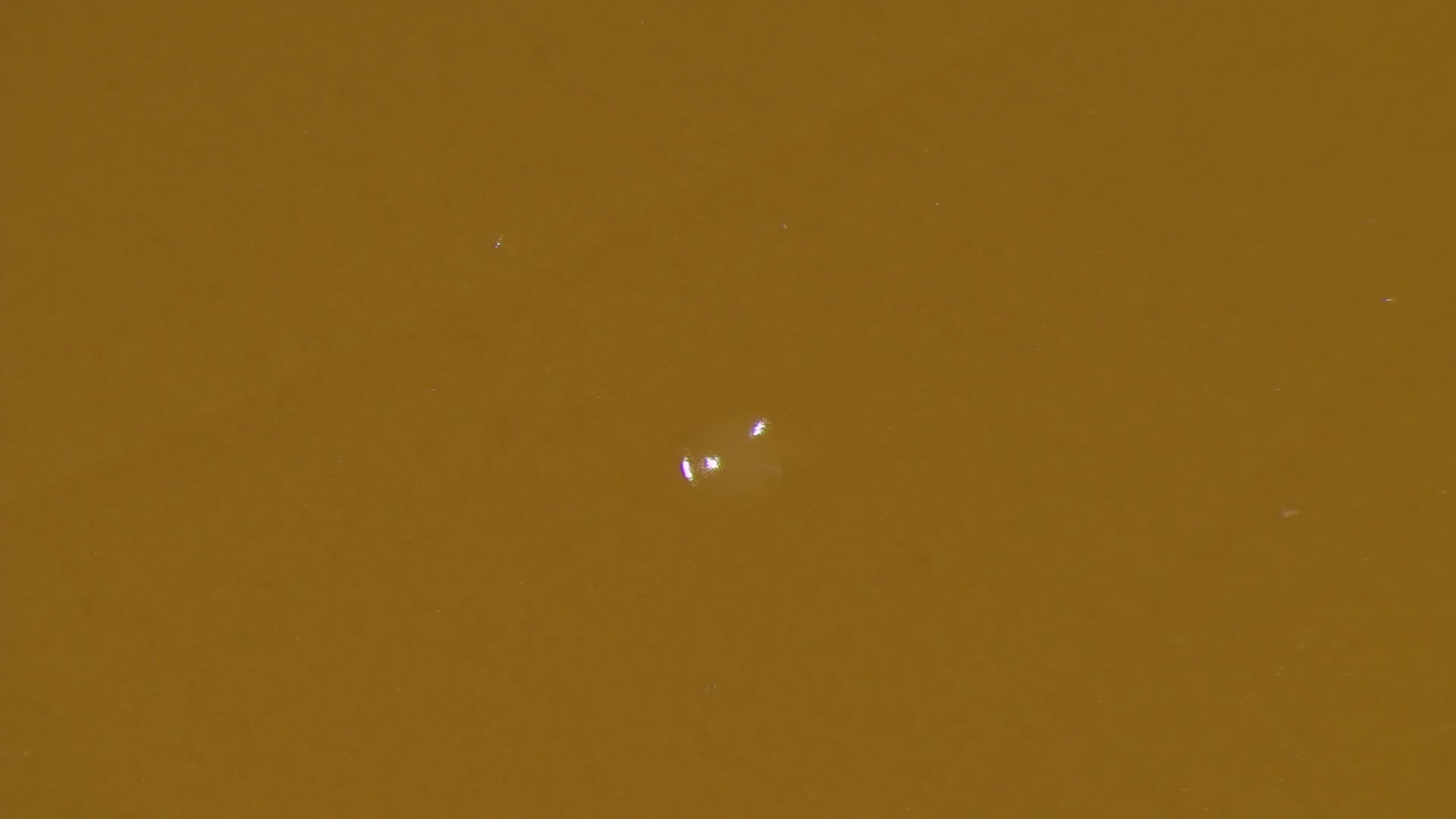

Supplement: Figure 3—figure supplement 1—source data 2. [file elife-90148-fig3-figsupp1-data2.zip › FigureS5_single_colonies_SN/BY4741_nutrient_rich_medium.jpg]

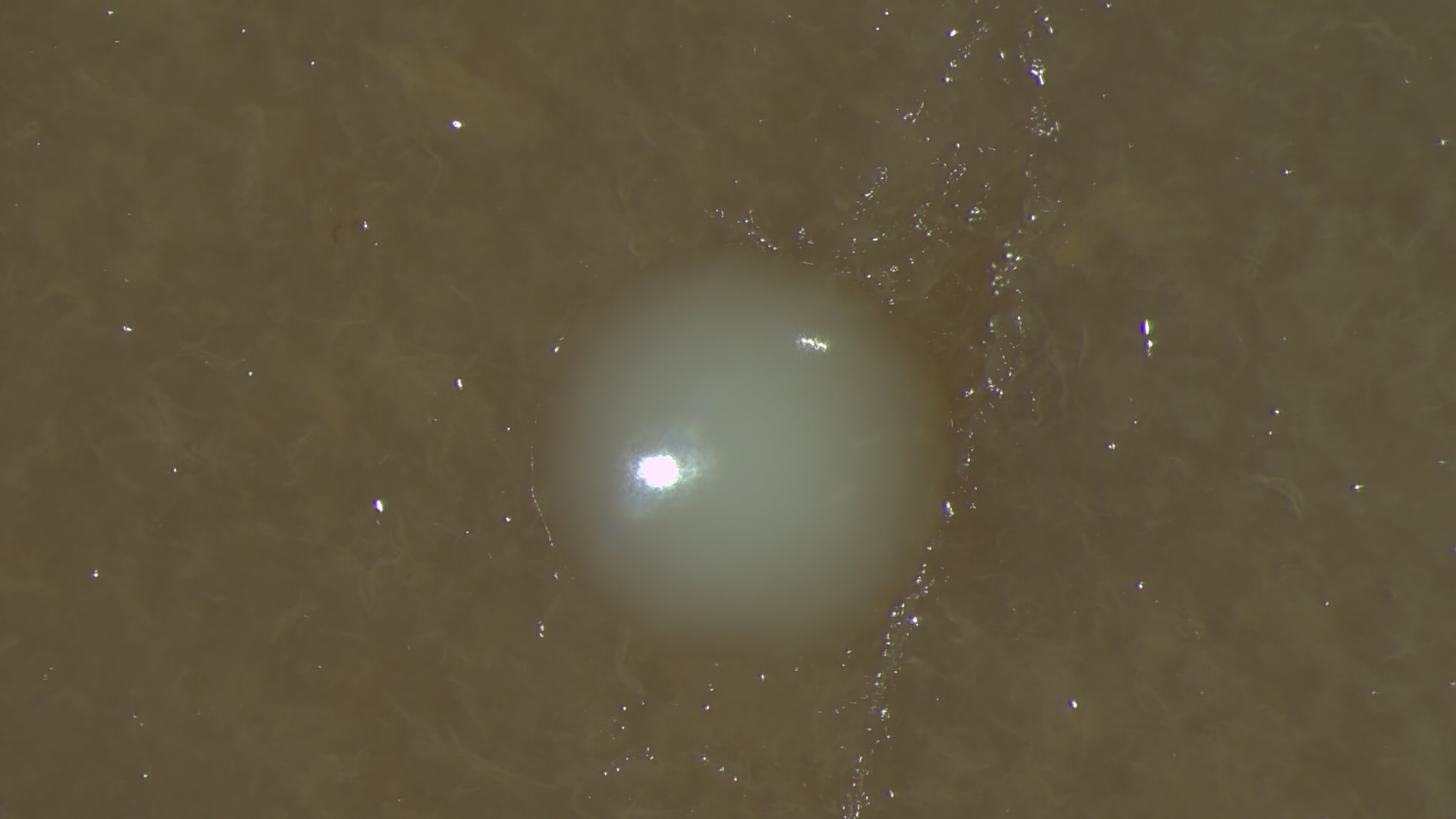

Supplement: Figure 3—figure supplement 1—source data 2. [file elife-90148-fig3-figsupp1-data2.zip › FigureS5_single_colonies_SN/H_uvarum_banana_agar.jpg]

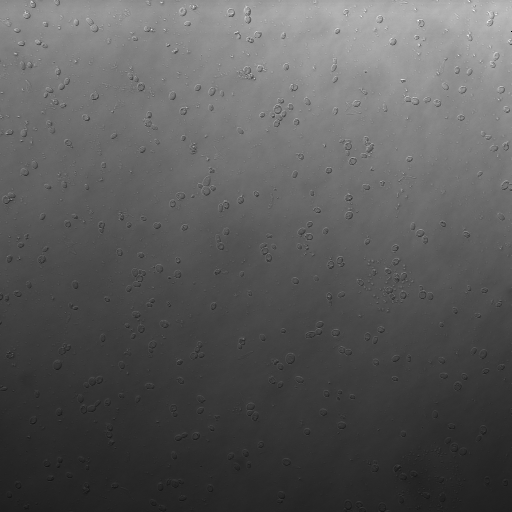

Supplement: Figure 6—source data 2. [file elife-90148-fig6-data2.zip › Figure6_phloxineB_images_SN/K_humilis_trans.tif]

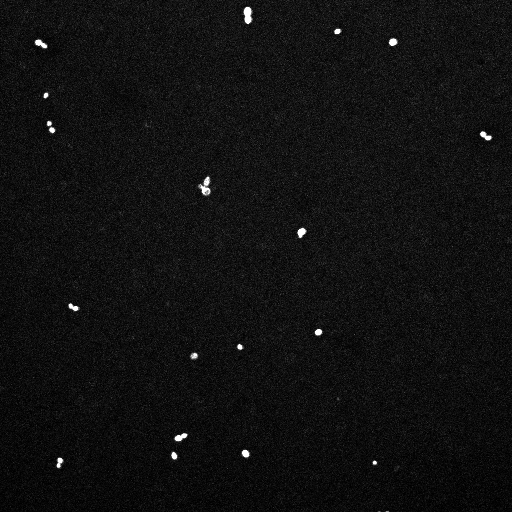

Supplement: Figure 6—source data 2. [file elife-90148-fig6-data2.zip › Figure6_phloxineB_images_SN/K_humilis_phloxineB.tif]

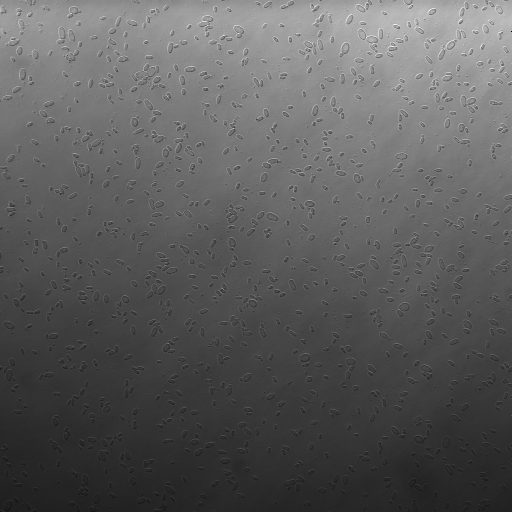

Supplement: Figure 6—source data 2. [file elife-90148-fig6-data2.zip › Figure6_phloxineB_images_SN/Pi_kluyveri_trans.tif]

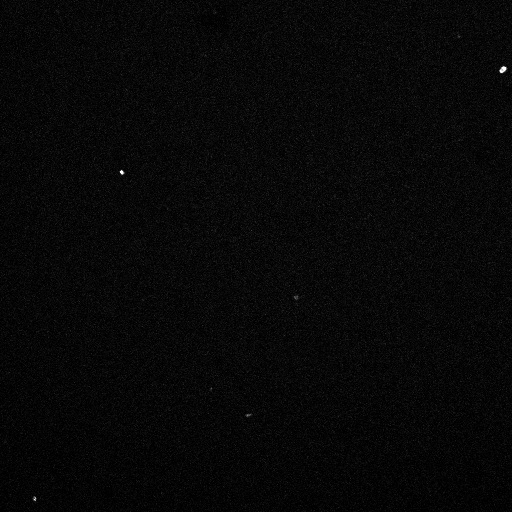

Supplement: Figure 6—source data 2. [file elife-90148-fig6-data2.zip › Figure6_phloxineB_images_SN/St_bacillaris_phloxineB.tif]

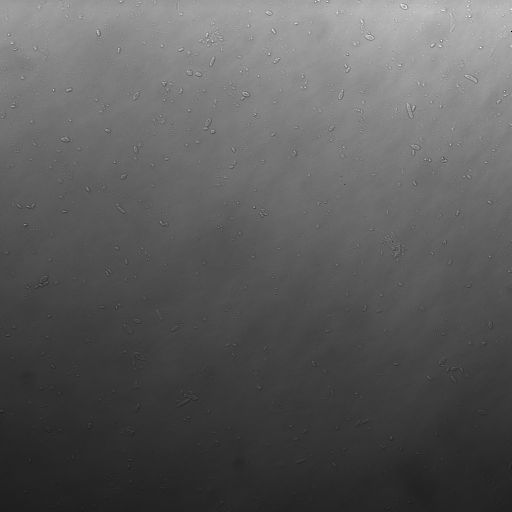

Supplement: Figure 6—source data 2. [file elife-90148-fig6-data2.zip › Figure6_phloxineB_images_SN/H_uvarum_trans.tif]

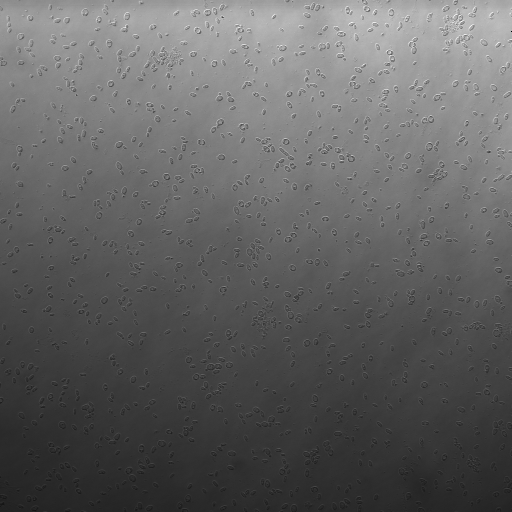

Supplement: Figure 6—source data 2. [file elife-90148-fig6-data2.zip › Figure6_phloxineB_images_SN/St_bacillaris_trans.tif]

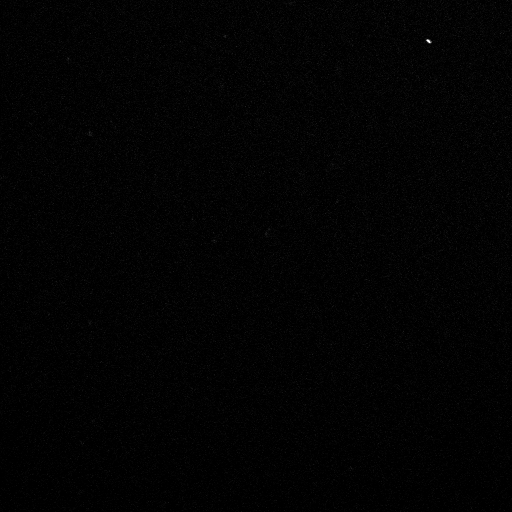

Supplement: Figure 6—source data 2. [file elife-90148-fig6-data2.zip › Figure6_phloxineB_images_SN/Pi_kluyveri_phloxineB.tif]

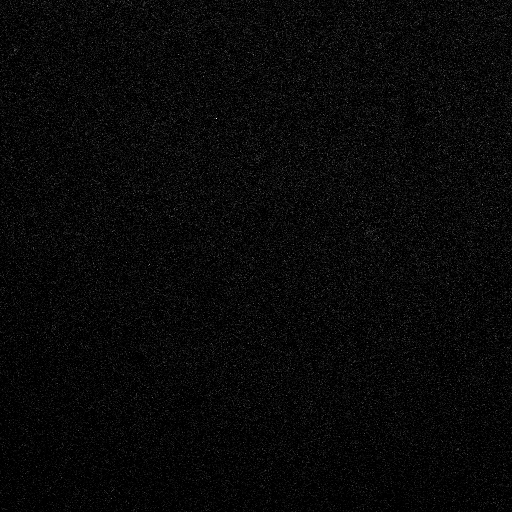

Supplement: Figure 6—source data 2. [file elife-90148-fig6-data2.zip › Figure6_phloxineB_images_SN/H_uvarum_phloxineB.tif]
